# PTB domain and leucine zipper motif 1 (APPL1) inhibits myocardial ischemia/hypoxia-reperfusion injury via inactivation of apoptotic protease activating factor-1 (APAF-1)/Caspase9 signaling pathway

**DOI:** 10.1080/21655979.2021.1954841

**Published:** 2021-07-24

**Authors:** Lina Bai, Junhua Yang, Hong Zhang, Wei Liao, Yunguang Cen

**Affiliations:** a Department of Cardiology, Tianjin Nankai Hospital, Tianjin, PR China; b Department of Medical Ultrasonics, Hainan General Hospital (Hainan Affiliated Hospital of Hainan Medical University), Haikou, PR China; c Center of Geriatrics, Hainan General Hospital (Hainan Affiliated Hospital of Hainan Medical University), Haikou, PR China

**Keywords:** APPL1, myocardial ischemia/hypoxia-reperfusion, apaf-1, caspase9

## Abstract

Myocardial ischemia/hypoxia-reperfusion injury mediates the progression of multiple cardiovascular diseases. It has been reported that knockdown of adaptor protein containing a PH domain, PTB domain and leucine zipper motif 1 (APPL1) is a significant factor for the progression of myocardial injury. However, the role of APPL1 in myocardial ischemia remains unclear. Hence, the aim of the present study was to investigate the specific mechanism underlying the role of APPL1 in myocardial ischemia.

In our study, the mRNA level of APPL1 was detected by quantitative real-time PCR (RT-qPCR). The expressions of APPL1, Apoptotic protease activating factor-1 (APAF-1), cleaved caspase9 and other inflammation- and apoptosis-related proteins were determined by western blotting. The secretion of inflammatory cytokines and lactate dehydrogenase (LDH) levels were measured by commercial assay kits. The H9C2 cell viability was analyzed by cell counting kit-8 (CCK-8) assay. The apoptosis rate of H9C2 cells was analyzed by TUNEL assay. The interaction between APPL1 and APAF-1/caspase9 was determined by Immunoprecipitation (IP).

Our findings demonstrated that APPL1 was low expressed in myocardial ischemia tissues and cells. APPL1 knockdown suppressed the viability of myocardial ischemia cells and aggravated hypoxia/reperfusion-induced LDH hypersecretion, inflammation and apoptosis. In addition, the overexpression of APPL1 induced inactivation of APAF-1/Caspase9 signaling pathway. Significantly, APAF1 inhibitor reversed the effect of APPL1 knockdown on viability, LDH secretion, inflammation and apoptosis.

We conclude that APPL1 inhibits myocardial ischemia/hypoxia-reperfusion injury via inactivation of APAF-1/Caspase9 signaling pathway. Hence, APPL1 may be a novel and effective target for the treatment of myocardial ischemia.

## GRAPHICAL ABSTRACT

**Figure:**
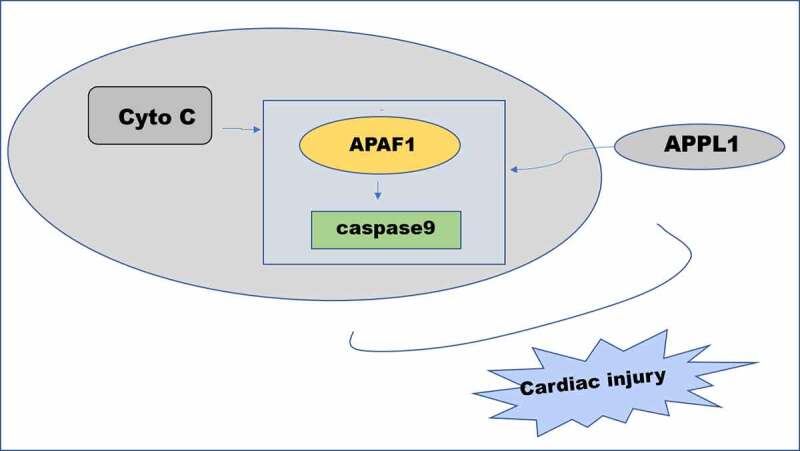

## Introduction

Myocardial ischemia is a severe pathological condition that mediates the progression of multiple cardiovascular diseases, including myocardial infarction, stroke, and peripheral vascular disease [1]. An early intervention to restore blood flow to the ischemic myocardium is a common strategy to salvage cardiomyocytes and limit infarct size [2,3]. However, after interruption of myocardial blood supply and oxygen supply in a short time, the reperfusion often results in paradoxical cardiomyocyte death and dysfunction, a phenomenon termed myocardial ischemia/hypoxia-reperfusion injury [4–6]. Myocardial ischemia/hypoxia-reperfusion injury usually causes massive myocardial cell apoptosis, which leads to myocardial infarct size s [7–11]. The cell death induced by myocardial ischemia/hypoxia-reperfusion injury involves intricate pathological mechanisms that do not render the detailed mechanism of myocardial ischemia/hypoxia-reperfusion injury fully illuminated.

Adaptor protein containing a PH domain, PTB domain and leucine zipper motif 1 (APPL1) is an important intracellular binding partner for Adiponectin Receptor (AdipoR) contributing to the pathogenesis of acute injury or fibrosis [12,13]. Previous study has reported that APPL1 upregulation attenuated late kidney fibrosis induced by ischemia reperfusion [14]. Moreover, adiponectin alleviated hypoxia/ischemia-induced neuronal apoptosis and brain atrophy through the activation of AdipoR1/APPL1 signaling pathway [15]. Of note, it has been reported that APPL1 knockdown partly abolished the protective effect of adiponectin on cardiomyocytes apoptosis induced by hypoxia/reoxygenation [16]. However, there are few studies on the role of APPL1 in myocardial ischemia. APAF-1 with a molecular weight of 130kD is involved in the intrinsic or mitochondrial pathway of apoptosis and, recruiting and activating procaspase9 to form apoptotic complex [17,18], the inhibition of which could ameliorate the I/R-induced injury. APPL1 is predicted to binding to caspase9 /APAF-1 by String database [19,20]. We predicted that the role of APPL1 in myocardial injury induced by ischemia-reperfusion could be related to caspase9 /APAF-1. Hence, the aim of the present study was to investigate the specific mechanism underlying the role of APPL1 in myocardial injury caused by ischemia/reperfusion and clarified whether caspase9 /APAF-1 is involved in the mechanism of APPL1 in myocardial ischemia/reperfusion model in vivo/vitro.

## Materials and methods

### An animal model of ischemia-reperfusion injury

Eight-week-old C57BL/6 mice (male, weight: 20–25 g, age: 6 weeks) were anesthetized by injection of sodium pentobarbital (60 mg/kg, i.p.) followed by tracheal intubation aided by a rodent ventilator. The mice were randomly divided into two groups: control (n = 6) and I/R (n = 6). By a standard surgical approach, ischemia-reperfusion injury (IR) was induced by 30 min of ischemia followed by 6 h of reperfusion injury [21]. Sham surgical procedures were performed on the control group. The mice were quickly decapitated, the brain tissue was removed and placed on ice, and the cerebellum and brainstem were removed to detect the corresponding indexes. The study was approved from the Ethics Committee of Hainan General Hospital.

## Cell culture and transfection

To establish a cellular hypoxia/reperfusion model (H/R), serum/glucose-free DMEM was used. H9C2 cells (rat embryonic-heart derived cell lines) in H/R group, obtained from the American Type Culture Collection, were cultured in anaerobic conditions (94% N_2_, 5% CO_2_, and 1% O_2_) and maintained at 37°C for 4 h [22]. The cells were then transferred to the normoxic incubator for 4 h to undergo reoxygenation. Cells under normoxic conditions served as a control.

Small interfering (si)-APPL1 (siRNA_APPL1-1: 5ʹ-CCGAAAGGCUCCAUACCUUTT-3ʹ; 5-TTGGCUUUCCGACCUAUGGAA-3. siRNA_APPL1-2, 5ʹ-GGAUUUAUGAUGCACAGAATT-3ʹ, 5ʹ-TTCCUAAAUACUACGUGUCUU-3ʹ. siRNA-NC: 5ʹ-UUCUCCGAACGUGUCACGUTT-3ʹ, 5ʹ-TTAAGAGGCUUGCACAGUGCA-3ʹ), the scramble siRNA plasmids overexpressing APPL1 (Ov-APPL1, 1 µg/ml) and its control (empty plasmids, Ov-NC, 1 µg/ml) were obtained from Shanghai GenePharma Co., Ltd. (Shanghai, China). The si-APPL1 (50 nM) and si-NC (50 nM) were transfected into H9C2 cells using Lipofectamine 2000 (Invitrogen, Carlsbad, CA) according to the manufacturer’s instructions. After 24 h, the cells were subjected to H/R treatment.

## Quantitative real-time PCR

The total RNA was extracted from heart tissues or H9C2 cells with Trizol reagent (Invitrogen, Carlsbad, CA, USA). After authentication of RNA quality and quantity, total RNA was reversely transcribed into complementary DNA using a Transcription kit (Titan One Tube RT-PCR, Roche) according to manufacturer’s protocols. A SYBR green qPCR assay kit (TaKaRa, Japan) was employed to amplify the expression level of APPL1 with GAPDH as the internal references. The PCR conditions consisted of predegeneration for 5 min at 95°C, and cycles of 40 at 95°C for 10s and at 60°C for 30s. The 2^﹣ΔΔCt^ method [23] was adopted to calculate the relative mRNA levels of APPL1. The primers used are as following: APPL1 Forward: 5ʹ- GCCACCAACAGCTCGAACCAG-3ʹ, Reverse: 5ʹ- TGTACGCCTGCCTCCTTGACC-3ʹ. GAPDH Forward: 5ʹ- AGGTCGGTGTGAACGGATTTG −3ʹ, Reverse: 5ʹ- GGGGTCGTTGATGGCAACA-3ʹ.

## Western blotting

The heart tissues and H9C2 cells of each group were lysed with RIPA lysis buffer (Beyotime, Shanghai, China) to extract the total protein. Then, 25 µg protein samples were fractionated by 10% SDS-PAGE and transferred onto PVDF membranes. Thereafter, the membranes were blocked, incubated at 4°C overnight with primary antibodies, and corresponding HRP‐labeled secondary antibody at room temperature for 2 h. Primary antibodies, including anti-APPL1 antibody (1:1000), anti-NF-κb p65 antibody (1:1000) and anti-Cox2 antibody (1:1000), anti-Bcl-2 antibody (1:1000), anti-Bax antibody (1:1000), anti-Cleaved PARP antibody (1:500), anti-APAF1 antibody (1:1000), anti- Cleaved caspase9 antibody (1:1000), anti-Caspase9 antibody (1:1000), were obtained from Cell Signaling Technology. Chemiluminescent (Immobilon ECL Ultra Western HRP) was used to develop color (Sigma-Aldrich) models. The protein bands were visualized using an automatic gel imaging analyzer. The intensity of the bands was quantified using Image Lab software and normalized to GAPDH expression.

## Hematoxylin-Eosin (HE) staining

The heart tissues were fixed with 4% neutral paraformaldehyde, washed with running water for 5 min after 48 h, dehydrated with gradient alcohol, and sliced into 3–4 sections (5 μm thick) using the paraffin slicing machine. Subsequently, the tissue sections were baked at 70°C for 60 min, followed by deparaffinization, hydration, HE staining, and sealed with neutral balsam. After HE staining, a light microscope was employed for histological observation.

## CCK-8 assay

H9C2 cells were plated into 96-well plates at a density of 5.0 × 10^3^/well. After 24 h of incubation, 10 μL of cell counting kit-8 (CCK-8) reagent was added to each well and cultured at 37°C for 1 h. The absorbance of each sample was measured at 450 nm using a Microplate Reader (Bio-Rad, Hercules, CA, USA). This experiment was repeated at least three independent times.

## Detection of LDH, TNF-α, IL-1β and IL-6

Transfected H9C2 cells were collected for detecting activities of lactate dehydrogenase (LDH) following the manufacturer’s instructions of LDH cytotoxicity assay kit (Beyotime, Shanghai, China). Additionally, the concentrations of inﬂammatory cytokines including TNF-α, IL-1β and IL-6 in supermatants of cells were determined by specific ELISA kits (Invitrogen, Carlsbad, CA, USA).

## TUNEL assay

To assess apoptosis rate of H9C2 cells, 4% paraformaldehyde was used to fix the cells. PBS containing 0.3% Triton X-100 was added to H9C2 cells. Finally, TUNEL solution (50 µl) was added for staining, and nuclear cells labeled positively were considered as apoptotic cells. The positive cells were detected by a fluorescence microscope (Nikon, Japan) at least five random separate fields. The percentage of apoptosis cells was calculated and analyzed.

## Immunoprecipitation assay (IP)

In order to further explore the mechanism of accelerating the inflammatory injury and apoptosis of cardiomyocytes after APPL1 silencing, it was predicted that APPL1 could combine with caspase9 /APAF-1 through String database (https://www.string-db.org/). Next, IP assay was used to explore their association. Cells were collected and lysed with IP lysis buffer containing protease inhibitor. After centrifugation at 12,000 × g at 4°C, anti-APAF1 or Caspase9 antibody (1 µg) was added into the supernatant on a rotating platform overnight at 4°C. Subsequently, 50 µl of SureBeads™ protein G magnetic beads (no. 1,614,023; Bio-Rad Laboratories, Inc., Hercules, CA, USA) were added into the above mixture at 4°C with gentle rotation for 4 h. The pellets were dissolved in 60 µl 1x electrophoresis sample buffer and boiled at 95°C for 5 min. Samples (30 µl) were analyzed by western blotting as aforementioned.

## Statistical analysis

All values are presented as the mean ± SEM and analyzed using GraphPad Prism® 5.0 (GraphPad Software, Inc., La Jolla, CA). One-way ANOVA followed by turkey’s post hoc test was performed to determine the differences in the means among multiple groups. *P* < 0.05 was considered statistically significant.

## Results

### APPL1 was low expressed in myocardial ischemia tissues and cells

**Figure 1.**
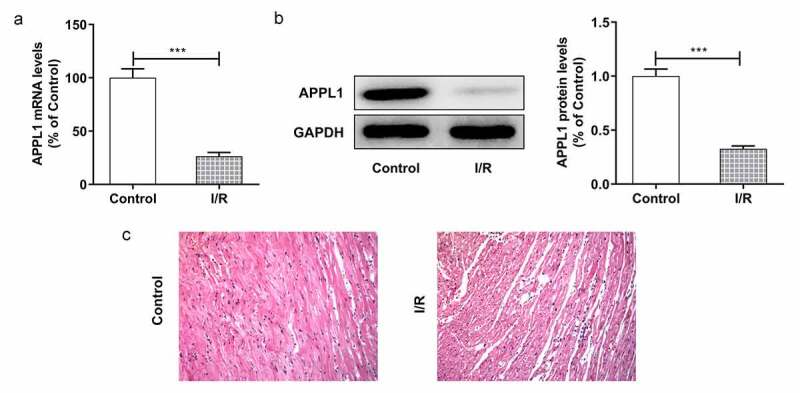
APPL1 expression level is low in myocardial ischemia tissues of mice. (Control: n = 6, H/R: n = 6) (a) The mRNA level of APPL1 in myocardial tissues was measured by Quantitative real-time PCR. (b) The APPL1 protein in myocardial tissues was determined by western blotting. (c) The pathological changes of myocardial tissues were observed by hematoxylin-Eosin (HE) staining. Error bars represent the mean ± SEM from three independent experiments. ***P < 0.001

**Figure 2.**
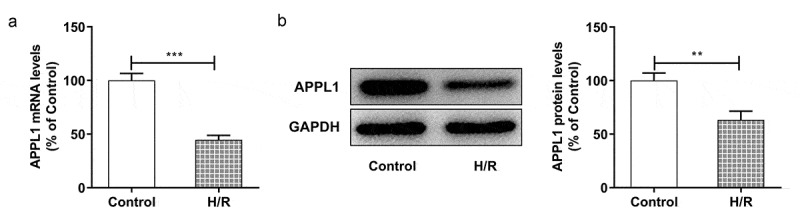
APPL1 was low expressed in H9C2 cells under H/R exposure (a) The mRNA level of APPL1 in myocardial ischemia cells was measured by Quantitative real-time PCR. (b) The APPL1 expression in myocardial ischemia cells was determined by western blotting. Error bars represent the mean ± SEM from three independent experiments. **P < 0.01, ***P < 0.001

To explore the role of APPL1 in myocardial ischemia/hypoxia-reperfusion injury, the APPL1 expression was measured by RT-qPCR and western blotting. As shown in Figure 1a&B, the mRNA and protein expression of APPL1 was significantly decreased in myocardial tissues in mice with I/R treatment compared to controls. Meanwhile, the histological changes were observed by HE staining. The results revealed that there were no abnormalities in control group, and the myocardial fibers were arranged orderly without enlarged necrotic gap. In I/R group, there were myocardial necrosis with an obviously enlarged gap (Figure 1c). Next, we wonder to know the role of APPL1 and uncover its mechanism in H/R-caused injury in H9c2 cells. Consistent with the observation in myocardial ischemia tissues, the results from RT-qPCR and western blotting showed that hypoxia-reperfusion treatment caused a reduction in APPL1 expression in H9C2 cells when compared with control group (Figure 2 A & B). These results demonstrated that APPL1 was low expressed in myocardial ischemia tissues and cells.

## APPL1 knockdown suppressed the viability of myocardial ischemia cells and aggravated LDH secretion

**Figure 3.**
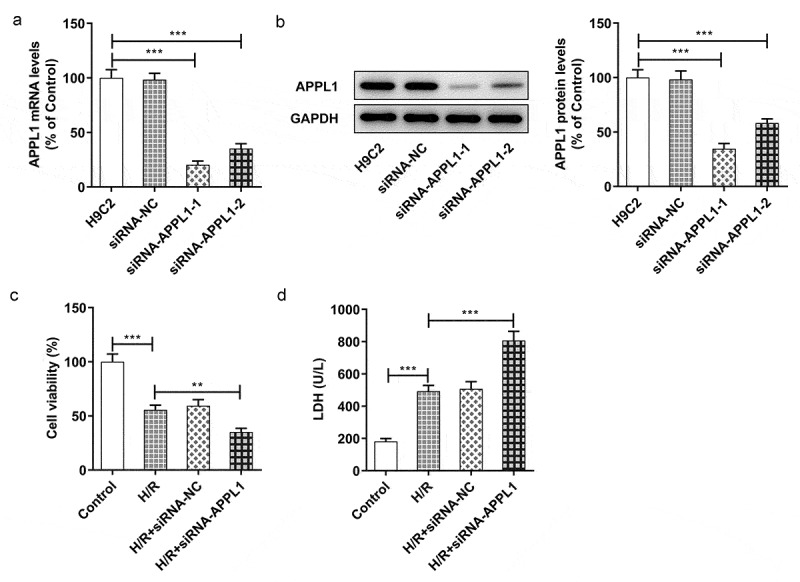
APPL1 knockdown suppressed the viability of H9C2 cells under H/R exposure, and aggravated LDH secretion. (a) The mRNA level of APPL1 in H9C2 cells was measured by Quantitative real-time PCR. (b) The APPL1 expression in H9C2 cells was determined by western blotting. (c) The H9C2 cell viability was detected by CCK8 assay. (d) The LDH level was analyzed by commercial assay kit. Error bars represent the mean ± SEM from three independent experiments. **P < 0.01, ***P < 0.001

To investigate the role of APPL1 in H9C2 cells under H/R exposure, the APPL1 knockdown was achieved by transfection of siRNA-APPL1. In Figure 3a&B, the APPL1 expression in siRNA-APPL1-1 group tended to be less than that in siRNA-APPL1-2 group. Hence, siRNA-APPL1-1 was selected for subsequent experiments. At the same time, the H9C2 cell viability was detected by CCK-8 assay As shown in Figure 3c, APPL1 knockdown suppressed viability of H9C2 cells with H/R treatment. Moreover, APPL1 knockdown induced hypersecretion of LDH in H9C2 cells with H/R treatment (Figure 3d). These results suggested that APPL1 knockdown suppressed viability of myocardial ischemia cells and aggravated LDH secretion.

## APPL1 knockdown aggravated hypoxia/reperfusion-induced inflammation and apoptosis

**Figure 4.**
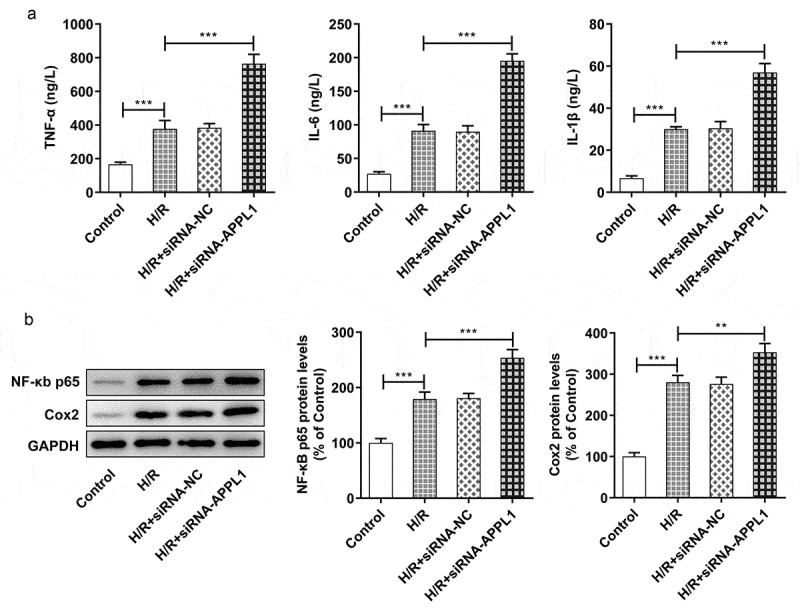
APPL1 knockdown aggravated hypoxia/reperfusion-induced inflammation in H9C2 cells. (a) The production of inflammatory cytokines including TNF-α, IL-1β and IL-6 was quantified with corresponding ELISA kit. (b) The expression levels of NF-κb p65 and Cox2 were determined by western blotting. Error bars represent the mean ± SEM from three independent experiments. **P < 0.01, ***P < 0.001

**Figure 5.**
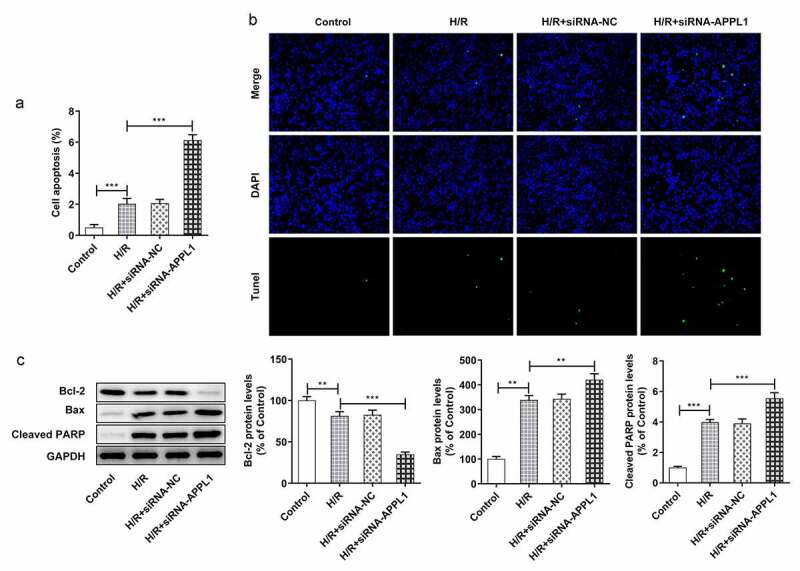
APPL1 knockdown aggravated hypoxia/reperfusion-induced apoptosis in H9C2 cells. (a-b) The H9C2 cell apoptosis was analyzed by TUNEL assay and quantification. (c) The expression levels of Bcl-2, Bax, cleaved PARP were determined by western blotting. Error bars represent the mean ± SEM from three independent experiments. **P < 0.01, ***P < 0.001

To further investigate the role of APPL1 in H9C2 cells challenged with H/R,, the effect of APPL1 knockdown on inflammation and cell apoptosis was analyzed. The inflammatory cytokines including TNF-α, IL-1β and IL-6 were measured with ELISA kits. As shown in Figure 4a, APPL1 knockdown significantly increased the concentration of TNF-α, IL-1β and IL-6 in H9C2 cells with H/R treatment, as well as in inflammation-related proteins including NF-κb p65 and Cox2 (Figure 4b). Furthermore, TUNEL assay was performed to determine the cell apoptosis rate. As shown in Figure 5a&B, APPL1 knockdown led to higher apoptosis rate of H9C2 cells with H/R treatment. Additionally, western blotting results presented that Bcl-2 expression (anti-apoptosis protein) was decreased, while Bax (pro-apoptosis protein) and Cleaved PARP expressions were increased in H9C2 cells from H/R+ siRNA-APPL1 compared to H/R group (Figure 5c). Thus, we concluded that APPL1 knockdown aggravated H/R-induced inflammation and apoptosis.

## The effect of APPL1 on APAF-1/Caspase9 signaling pathway

**Figure 6.**
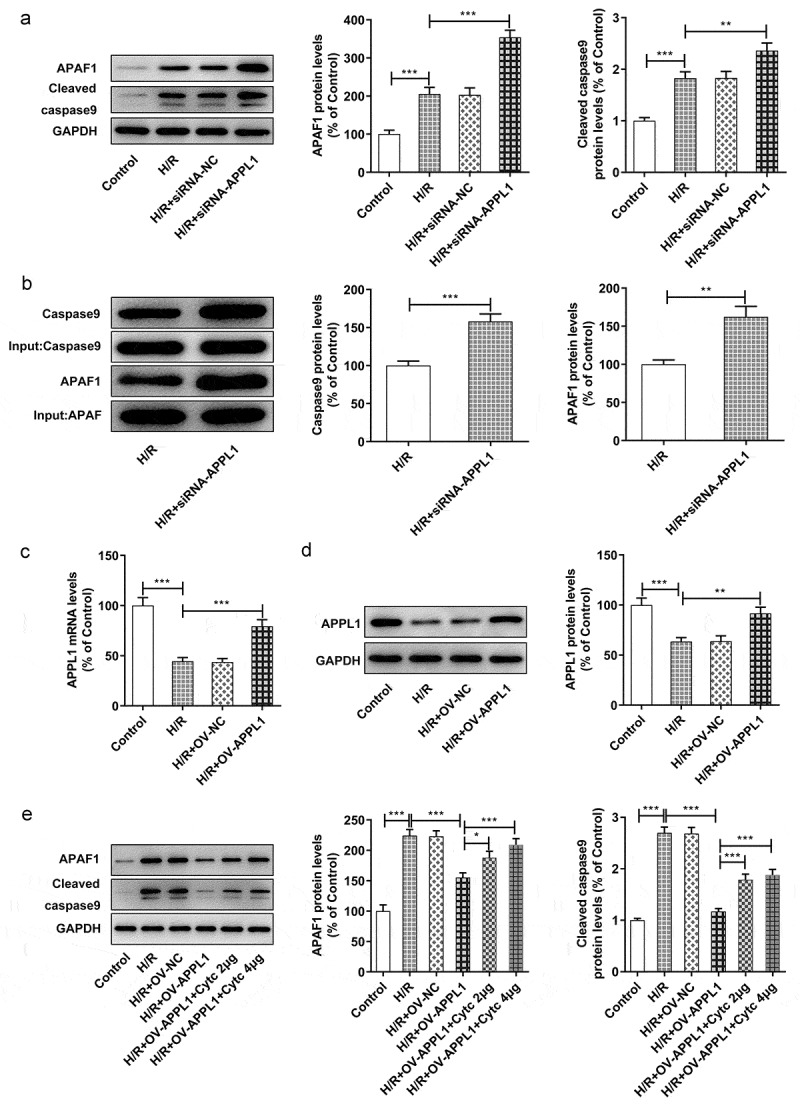
The effect of APPL1 on APAF-1/Caspase9 signaling pathway in H9C2 cells with H/R stimulation. (a) The expression levels of APAF-1, Cleaved caspase9/caspase9 were determined by western blotting. (b) The interaction between APPL1 and APAF-1/Caspase9 was verified by immunoprecipitation (IP). Caspase9 was immunoprecipitated (IP) from H9C2 cells exposed to H/R stimulation with or without siRNA-APPL1 transfection, and the immunoprecipitates or supernatant (Input) were immunoblotted for Caspase9 and APAF-1. (c) The mRNA level of APPL1 in H9C2 cells was measured by Quantitative real-time PCR. (d) The APPL1 expression in H9C2 cells was determined by western blotting. (e) The expression levels of APAF-1, Cleaved caspase9/caspase9 were determined by western blotting. Error bars represent the mean ± SEM from three independent experiments. *P < 0.05, **P < 0.01, ***P < 0.001

To investigate the detailed mechanism underlying the role of APPL1 in H9C2 cells subjected to H/R treatment, the interaction in APPL1 and caspase9/APAF-1 were predicted using String database. The western blotting results demonstrated that APPL1 knockdown enhanced the expressions of APAF-1 and Cleaved caspase9 in H9C2 cells with H/R treatment (Figure 6a). Next, the immunoprecipitates of caspase9 were detected with antibody to caspase9 or APAF-1. APAF-1 is co-immunoprecipitated with caspase9 from H9C2 cells under H/R treatment with or without siRNA-APPL1 transfection. Furthermore, APPL1 silencing significantly increased the protein levels of APAF-1 and caspase9 in (Figure 6b). IP results suggested that APPL1silencing can promote the interaction of caspase9 and APAF-1 (Figure 6b). In Figure 6c&D, the results from Western Blotting and RT-PCR proved the high transfection efficiency of OV-APPL1. APPL1 overexpression markedly induced the inactivation of APAF-1/Caspase9 signaling pathway, while the treatment of recombinant cytochrome C (Cytoc) abolished the inhibitive effect of APPL1 overexpression on APAF-1 and Cleaved caspase9 expression (Figure 6e). These results implied that APAF-1/Caspase9 signaling pathway is mediated by the role of APPL1 in myocardial ischemia.

**APAF1 inhibitor reversed the effect of APPL1 knockdown on the cell viability, LDH secretion, inflammation and apoptosis**

**Figure 7.**
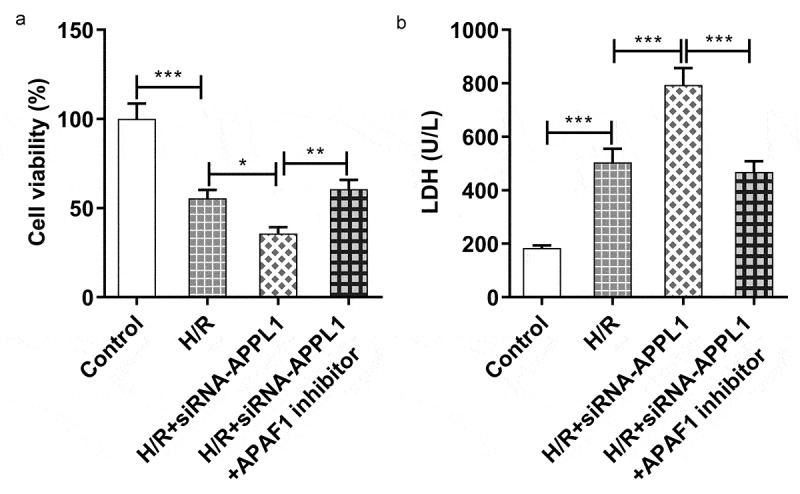
APAF1 inhibitor reversed the effect of APPL1 knockdown on viability and LDH secretion in H9C2 cells under H/R exposure. (a) The H9C2 cell viability was detected by CCK8 assay. (b) The LDH level was analyzed by commercial assay kit. Error bars represent the mean ± SEM from three independent experiments. *P < 0.05, **P < 0.01, ***P < 0.001

**Figure 8.**
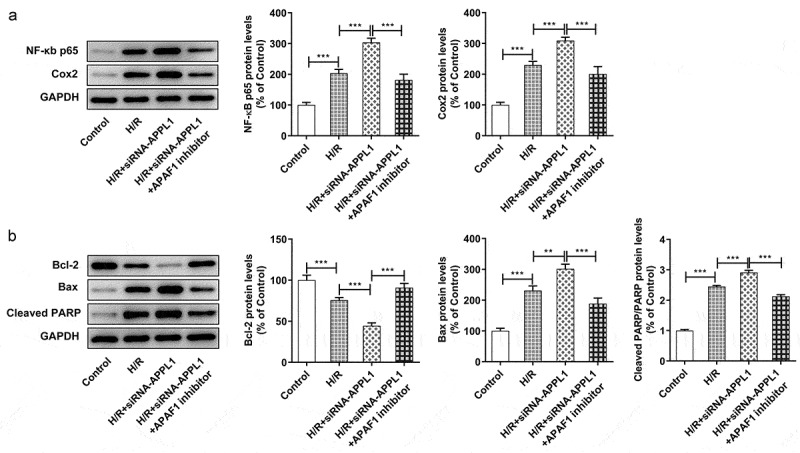
APAF1 inhibitor reversed the effect of APPL1 knockdown on inflammation and apoptosis in H9C2 cells under H/R challenge. (a) The expression levels of NF-κb p65 and Cox2 were determined by western blotting. (b) The expression levels of Bcl-2, Bax, cleaved PARP were quantified by western blotting. Error bars represent the mean ± SEM from three independent experiments. **P < 0.01, ***P < 0.001

To confirm whether APAF-1/Caspase9 signaling pathway mediated the role of APPL1 in H9C2 cells exposed to H/R,, APAF1 inhibitor (ZYZ-488) was used in further study. As shown in Figure 7a, the H9C2 cell viability was elevated by APAF1 inhibitor treatment. Meanwhile, APAF1 inhibitor remarkably depressed the LDH secretion in H9C2 cells transfected with siRNA-APPL1 (Figure 7b). Furthermore, ZYZ-488 treatment inhibited the expression of inflammation-related proteins (NF-κB p65, Cox2) and apoptosis-related proteins (Bcl-2, Bax, cleaved PARP) in H9C2 cells from H/R+ siRNA-APPL1+ APAF1 inhibition group, compared to H/R+ siRNA-APPL1 group (Figure 8a&B). These results indicated that APAF1 inhibitor reversed the effect of APPL1 knockdown on viability, LDH secretion, inflammation and apoptosis.

## Discussion

Myocardial ischemia-reperfusion (I/R) injury is a major stimulative factor for the morbidity and mortality related to coronary artery diseases [24]. Emerging evidence has implied that APPL1 was involved in the pathology of renal ischemia and brain ischemia [14,15]. However, it is unclear whether APPL1 could contribute to the onset and development of myocardial ischemia. Hence, the present study aimed to explore the role of APPL1 in myocardial ischemia.

In our study, the APPL1 expression was significantly reduced in cellular H/R model and the myocardial tissue of I/R injury mice model, suggesting APPL1 was closely related to pathogenesis of myocardial ischemia. Lactate dehydrogenase (LDH) level was remarkably increased at the end of ischemia and continued to be at aberrantly high levels throughout reperfusion progression [25]. LDH has also emerged as a reliable predictor to estimate the toxic potential at the cellular level [26]. APPL1 knockdown significantly suppressed H9C2 cell viability and aggravated LDH secretion, suggesting that APPL1 may protect myocardial cells against H/R treatment-induced cell injury. To further confirm the role of APPL1 in myocardial ischemia, the inflammation and apoptosis were evaluated by further assays. NF-κB is implicated in the expression of many downstream proinflammatory genes, such as Cox-2, TNF-α, IL-1β, and IL-6, which are involved in the inflammation response in myocardial infarction and myocardial ischemia/reperfusion [27,28]. The study found that the protein levels of Cox-2, NF-κB p65, TNF-α, IL-1β and IL-6, were significantly increased after H/R induction and further increased by APPL1 silencing. Furthermore, their levels were able to be reversed by the addition of APAF-1 inhibitor, which implied that APPL1 and caspase9/APAF-1 affected inflammation induced by H/R possibly related to NF-κB pathway. Inflammatory response induced by I/R stimulation is one of the most important elements in myocardial I/R injury, which plays a crucial role in the regulation of myocyte autophagy and apoptosis [29–31]. Cardiomyocyte apoptosis is an important remodeling event contributing to heart failure [16]. The ensuing finding that APPL1 knockdown led to higher cell apoptosis rates and increased the protein levels of pro-apoptotic-proteins, Bax and cleaved PARP, and reduced anti-apoptosis protein Bcl-2 protein levels that participated in the apoptosis induced by myocardial ischemia/reperfusion [32,33]. It was reported that the inhibition of PARP activity could keep ischemia–reperfusion injury in bay [34]. Thus, the results prompted the recognition that APPL1 can protect myocardial cells against H/R treatment-induced inflammatory response and cell apoptosis.

To investigate the detailed mechanism underlying the role of APPL1 in myocardial ischemia, the interaction between APPL1 and caspase9/APAF-1 was predicted and demonstrated by our findings. Furthermore, APPL1 exerted negatively regulative effect on the expressions of APAF1 and cleaved caspase9, which implied that APAF-1/Caspase9 signaling pathway may mediate the role of APPL1 in myocardial ischemia. It has been reported that APAF-1 was involved in the protective effect of caspase recruitment domain-containing protein 9 on cardiomyocytes from apoptosis following myocardial I/R injury in vivo and in vitro [19]. The APAF-1 inhibitor attenuated cardiomyocyte apoptosis via disturbing procaspase-9 recruitment by APAF-1 [15]. Moreover, APAF-1-interacting protein plays cardioprotective roles in myocardial infarction [35]. Subsequently, APAF1 inhibitor ZYZ-488 was used to further validate whether APAF-1/Caspase9 signaling pathway mediated the cardioprotective role of APPL1 in I/R injury. The following results indicated that APAF1 inhibitor markedly enhanced cell viability and inhibited LDH secretion, inflammation and cell apoptosis. Collectively, these data provided evidence that APPL1 inhibits myocardial I/R injury via inactivation of APAF-1/Caspase9 signaling pathway.

## Conclusion

Taken together, APPL1 knockdown suppressed the viability of myocardial ischemia cells and aggravated hypoxia/reperfusion-induced LDH hypersecretion, inflammation and apoptosis, which was, to our interest, abrogated by APAF1 inhibitor. Thus, APPL1 inhibits myocardial I/R injury via inactivation of APAF-1/Caspase9 signaling pathway. Hence, APPL1 may be presented as a novel and effective target for the treatment of myocardial ischemia.

## Highlights

1. APPL1 was involved in myocardial injury induced by hypoxia/reperfusion.
2. The inflammation and apoptosis caused by hypoxia/reperfusion could be regulated through APPL1 via APAF-1/Caspase9 signaling pathway.
3. APPL1 affected the binding of APAF-1 and Caspase9.

## References

[1] Hentia C, Rizzato A, Camporesi E, et al. Sﺅ√ndesc D and Bosco G. An overview of protective strategies against ischemia/reperfusion injury: the role of hyperbaric oxygen preconditioning. Brain Behav. 2018;8:e00959. 29761012 10.1002/brb3.959 PMC5943756

[2] Duan L, Liang C, Li X, et al. Wu N and Jia D. Lycopene restores the effect of ischemic postconditioning on myocardial ischemiaﻗ°∞reperfusion injury in hypercholesterolemic rats. Int J Mol Med. 2019;43:2451–2461. 31017253 10.3892/ijmm.2019.4166 PMC6488174

[3] Paradies V, Chan MHH, Hausenloy DJ. Strategies for Reducing Myocardial Infarct Size Following STEMI. In: Watson TJ, Ong PJL, Tcheng JE, editors. Primary Angioplasty: a Practical Guide. Singapore: 2018, The Author(s); 2018. p. 307–322. 31314426

[4] Jubair S, Li J, Dehlin HM, et al. Levick SP and Janicki JS. Substance P induces cardioprotection in ischemia-reperfusion via activation of AKT. Am J Physiol Heart Circ Physiol. 2015;309(4):H676–684. 26071541 10.1152/ajpheart.00200.2015 PMC4537946

[5] Pu Y, Wu D. Lu X and Yang L. Effects of GCN2/eIF2ﺧ١ on myocardial ischemia/hypoxia reperfusion and myocardial cells injury. Am J Transl Res. 2019;11:5586–5598. PMC6789277 31632531

[6] Thind GS, Agrawal PR, Hirsh B, et al. Mechanisms of myocardial ischemia-reperfusion injury and the cytoprotective role of minocycline: scope and limitations. Future Cardiol. 2015;11(1):61–76. 25606703 10.2217/fca.14.76

[7] Martin Del Campo SE, Latchana N, Levine KM, et al. 3rd. MiR-21 enhances melanoma invasiveness via inhibition of tissue inhibitor of metalloproteinases 3 expression: in vivo effects of MiR-21 inhibitor. PLoS One. 2015;10(1): e0115919. 10.1371/journal.pone.0115919 PMC4294659 25587717

[8] Chen L, Zhang D, Yu L, et al. MIAT reduces apoptosis of cardiomyocytes after ischemia/reperfusion injury. Bioengineered. 2019;10(1):121–132. 30971184 10.1080/21655979.2019.1605812 PMC6527071

[9] Jiao H, Chen R, Jiang Z, et al. miR-22 protect PC12 from ischemia/reperfusion-induced injury by targeting p53 upregulated modulator of apoptosis (PUMA). Bioengineered. 2020;11(1):209–218. 32065044 10.1080/21655979.2020.1729321 PMC7039629

[10] Liu K, Zhao D, Wang D. LINC00528 regulates myocardial infarction by targeting the miR-143-3p/COX-2 axis. Bioengineered. 2020;11(1):11–18. 31833800 10.1080/21655979.2019.1704535 PMC6961595

[11] Mao S, Tian S, Luo X, et al. Overexpression of PLK1 relieved the myocardial ischemia-reperfusion injury of rats through inducing the mitophagy and regulating the p-AMPK/FUNDC1 axis. Bioengineered. 2021;12(1):2676–2687. 34115550 10.1080/21655979.2021.1938500 PMC8806532

[12] Artimani T, Najafi R. APPL1 as an important regulator of insulin and adiponectin-signaling pathways in the PCOS: a narrative review. Cell Biol Int. 2020;44(8):1577–1587. 32339379 10.1002/cbin.11367

[13] Fang H, Judd RL. Adiponectin Regulation and Function. Compr Physiol. 2018;8:1031–1063. 29978896 10.1002/cphy.c170046

[14] Hongtao C, Youling F, Fang H, et al. Curcumin alleviates ischemia reperfusion-induced late kidney fibrosis through the APPL1/Akt signaling pathway. J Cell Physiol. 2018;233(11):8588–8596. 29741772 10.1002/jcp.26536

[15] Xu N, Zhang Y, Doycheva DM, et al. Adiponectin attenuates neuronal apoptosis induced by hypoxia-ischemia via the activation of AdipoR1/APPL1/LKB1/AMPK pathway in neonatal rats. Neuropharmacology. 2018;133:415–428. 29486166 10.1016/j.neuropharm.2018.02.024

[16] Park M, Youn B, Zheng X-L, et al. Globular adiponectin, acting via AdipoR1/APPL1, protects H9c2 cells from hypoxia/reoxygenation-induced apoptosis. PLoS One. 2011;6(4):e19143. 21552570 10.1371/journal.pone.0019143 PMC3084258

[17] Shakeri R, Kheirollahi A, Davoodi J. Apaf-1: regulation and function in cell death. Biochimie. 2017;135:111–125. 28192157 10.1016/j.biochi.2017.02.001

[18] Ferraro E, Pesaresi MG, De Zio D, et al. Apaf1 plays a pro-survival role by regulating centrosome morphology and function. J Cell Sci. 2011;124(20):3450–3463. 21984814 10.1242/jcs.086298

[19] Li Y, Liang P, Jiang B, et al. Liu Z and Xiao X. CARD9 inhibits mitochondria-dependent apoptosis of cardiomyocytes under oxidative stress via interacting with Apaf-1. Free Radic Biol Med. 2019;141:172–181. 31212066 10.1016/j.freeradbiomed.2019.06.017

[20] Wang Y, Zhang Q, Zhong L, et al. Apoptotic Protease Activating Factor-1 Inhibitor Mitigates Myocardial Ischemia Injury via Disturbing Procaspase-9 Recruitment by Apaf-1. Oxid Med Cell Longev. 2017;2017:9747296. 29279737 10.1155/2017/9747296 PMC5723966

[21] Fang J, Hu F, Ke D, et al. N,N-dimethylsphingosine attenuates myocardial ischemia–reperfusion injury by recruiting regulatory T cells through PI3K/Akt pathway in mice. Basic Res Cardiol. 2016;111(3):32. 27048490 10.1007/s00395-016-0548-3

[22] Li W, Li W, Leng Y, et al. Ferroptosis Is Involved in Diabetes Myocardial Ischemia/Reperfusion Injury Through Endoplasmic Reticulum Stress. DNA Cell Biol. 2020;39(2):210–225. 31809190 10.1089/dna.2019.5097

[23] Livak KJ, Schmittgen TD. Analysis of relative gene expression data using real-time quantitative PCR and the 2(-Delta Delta C(T)) Method. Methods. 2001;25(4):402–408. 11846609 10.1006/meth.2001.1262

[24] Li W, Li Y, Chu Y, et al. PLCE1 promotes myocardial ischemia-reperfusion injury in H/R H9c2 cells and I/R rats by promoting inflammation. Biosci Rep. 2019;39(7). 10.1042/BSR20181613 PMC6609553 31217261

[25] CaﺅŸlayan F, CaﺅŸlayan O, Gunel E, et al. Intestinal ischemia-reperfusion and plasma enzyme levels. Pediatr Surg Int. 2002;18(4):255–257. 12021974 10.1007/s003830100666

[26] Zou Y, Kim D, Yagi M, et al. Application of LDH-release assay to cellular-level evaluation of the toxic potential of harmful algal species. Biosci Biotechnol Biochem. 2013;77(2):345–352. 23391929 10.1271/bbb.120764

[27] Sue Y-M, Cheng C-F, Chang -C-C, et al. Antioxidation and anti-inflammation by haem oxygenase-1 contribute to protection by tetramethylpyrazine against gentamicin-induced apoptosis in murine renal tubular cells. Nephrol Dial Transplant. 2008;24(3):769–777. 18842672 10.1093/ndt/gfn545

[28] Viswanadha VP, Dhivya V, Beeraka NM, et al. The protective effect of piperine against isoproterenol-induced inflammation in experimental models of myocardial toxicity. Eur J Pharmacol. 2020;885:173524. 32882215 10.1016/j.ejphar.2020.173524

[29] Gan J, Qian W, Lin S. Umbelliferone Alleviates Myocardial Ischemia: the Role of Inflammation and Apoptosis. Inflammation. 2018;41(2):464–473. 29188498 10.1007/s10753-017-0702-6

[30] Wang X, Guo Z, Ding Z, et al. Inflammation, Autophagy, and Apoptosis After Myocardial Infarction. J Am Heart Assoc. 2018;7(9):e008024.. 10.1161/JAHA.117.008024 PMC6015297 29680826

[31] Yao L, Chen H, Wu Q, et al. Hydrogen-rich saline alleviates inflammation and apoptosis in myocardial I/Rﺁ injury via PINK-mediated autophagy. Int J Mol Med. 2019;44:1048–1062. 31524220 10.3892/ijmm.2019.4264 PMC6657957

[32] Du Toit EF, Tai WS, Cox A, et al. Synergistic effects of low-level stress and a Western diet on metabolic homeostasis, mood, and myocardial ischemic tolerance. Am J Physiol Regul Integr Comp Physiol. 2020;319(3):R347–r357. 32755463 10.1152/ajpregu.00322.2019

[33] Zhang W, Zhang Y, Ding K, et al. Involvement of JNK1/2-NF-κBp65 in the regulation of HMGB2 in myocardial ischemia/reperfusion-induced apoptosis in human AC16 cardiomyocytes. Biomed Pharmacother. 2018;106:1063–1071. 30119172 10.1016/j.biopha.2018.07.015

[34] Lv S, Ju C, Peng J, et al. 25-Hydroxycholesterol protects against myocardial ischemia-reperfusion injury via inhibiting PARP activity. Int J Biol Sci. 2020;16(2):298–308. 31929757 10.7150/ijbs.35075 PMC6949155

[35] Lim B, Jung K, Gwon Y, et al. Cardioprotective role of APIP in myocardial infarction through ADORA2B. Cell Death Dis. 2019;10(7):511. 31263105 10.1038/s41419-019-1746-3 PMC6602929

